# Recent advances on innate immune pathways related to host–parasite cross-talk in cystic and alveolar echinococcosis

**DOI:** 10.1186/s13071-020-04103-4

**Published:** 2020-05-06

**Authors:** Nayer Mehdizad Bakhtiar, Adel Spotin, Mahmoud Mahami-Oskouei, Ehsan Ahmadpour, Ali Rostami

**Affiliations:** 1 Department of Parasitology and Mycology, Faculty of Medicine, Tabriz University of Medical Sciences, Tabriz, Iran; 2 Immunology Research Center, Tabriz University of Medical Sciences, Tabriz, Iran; 3 Student Research Committee, Tabriz University of Medical Sciences, Tabriz, Iran; 4 Infectious and Tropical Disease Research Center, Tabriz University of Medical Sciences, Tabriz, Iran; 5 Infectious Diseases and Tropical Medicine Research Center, Health Research Institute, Babol University of Medical Sciences, Babol, Iran

**Keywords:** Apoptosis, *Echinococcus granulosus*, *Echinococcus multilocularis*, Inflammasome, Innate immunity, Toll-like receptors

## Abstract

Cystic echinococcosis (CE) and alveolar echinococcosis (AE) are life-threatening parasitic infections worldwide caused by *Echinococcus granulosus* (*sensu lato*) and *E. multilocularis*, respectively. Very little is known about the factors affecting innate susceptibility and resistance to infection with *Echinococcus* spp. Although benzimidazolic drugs against CE and AE have definitively improved the treatment of these cestodes; however, the lack of successful control campaigns, including the EG95 vaccine, at a continental level indicates the importance of generating novel therapies. This review represents an update on the latest developments in the regulatory functions of innate immune pathways such as apoptosis, toll-like receptors (TLRs), and inflammasomes against CE and AE. We suggest that apoptosis can reciprocally play a bi-functional role among the host-*Echinococcus* metabolite relationships in suppressive and survival mechanisms of CE. Based on the available information, further studies are needed to determine whether the orchestrated *in silico* strategy for designing inhibitors and interfering RNA against anti-apoptotic proteins and TLRs would be effective to improve new treatments as well as therapeutic vaccines against the *E. granulosus* and *E. multilocularis.*
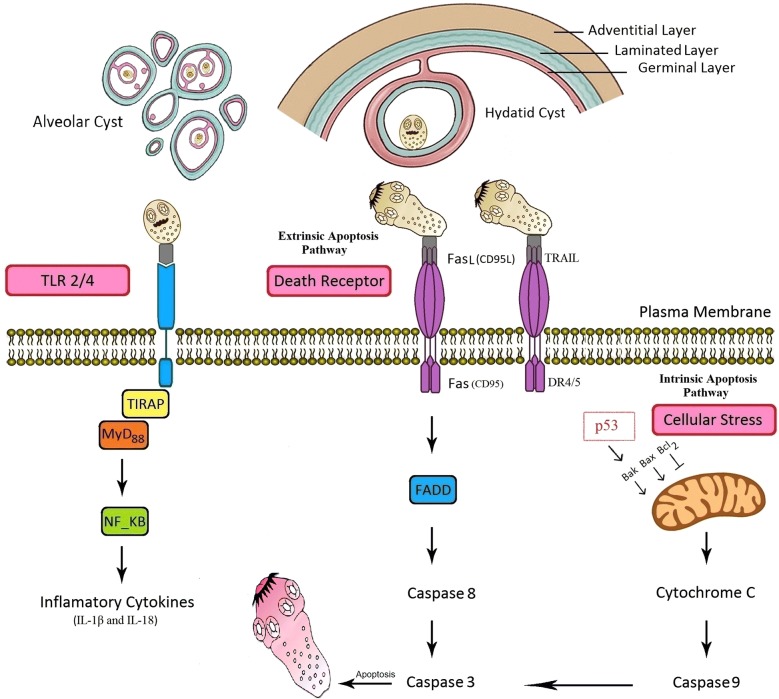

## Background

Cystic echinococcosis (CE) and alveolar echinococcosis (AE) are cosmopolitan neglected parasitic diseases caused by infection with the metacestodes of the *Echinococcus granulosus* (*sensu lato*) and *Echinococcus multilocularis*, respectively, which threaten human health worldwide [1, 2]. Adult tapeworms reside in the small bowel of canine definitive hosts and produce infectious eggs [2]. Humans as an incidental dead-end host are infected by the larvae after the ingestion of eggs [3, 4]. Following oral uptake of infectious eggs, the active oncosphere migrates through the intestinal wall and enters the bloodstream developing into cysts (CE) or infiltrative vesicles (AE) mainly in the liver. The growth of AE vesicles, similar to cancer progression, is steady, and they consist of an inner cellular germinal layer (GL) and an outer acellular carbohydrate-rich laminated layer (LL). CE cysts grow slowly, and the GL is supported by the laminated and adventitial (fibrous) layers [3, 5, 6]. It has been suggested that the GL and LL play a pivotal role in stimulating the innate immune responses in the host–parasite relationships due to the accumulation of various effective antigens and molecules [7–10]. Amri & Touil-Boukoffa [11] have indicated that the LL protects *E. granulosus* survival against the inducible nitric oxide (NO) synthase response through upregulation of the host arginase pathway. Furthermore, Zeghir-Bouteldja et al. [12] have shown that the production of NO species by activated macrophages plays a crucial role in preventing the dissemination of hydatid cyst layers (LL and GL).

Recent advances in immunological findings have disclosed new approaches regarding the innate immune responses generated during the establishment of CE/AE; however, various features of the *Echinococcus*-host interplay have not yet been widely investigated [13]. The generation of efficient vaccines against human CE and AE has been hampered, because of the intricacy of their life-cycles, the various immunomodulatory functions and also difficulties to implement effective control campaigns, including the EG95 vaccine at a continental level [14]. It has been well documented that helminth infections can stimulate the host protective type 2 immune responses [15]. The host immune system against CE is classically divided into the adaptive and innate response [15]. Innate immunity is the first line of defense against various parasites [16] that can recognize pathogen-associated molecules patterns (PAMPs), *via* pattern-recognition receptors (PRRs), such as toll-like receptors (TLRs) and nucleotide-binding oligomerization domain (NOD)-like receptors (NLRs). These receptors are expressed by the host innate immune cells, including macrophages, neutrophils, endothelial cells, dendritic cells (DCs) and lymphocytes, which modulate immune responses through different mechanisms for host defense [16–18]. Innate immunity in response to helminths is orchestrated during the stimulation of host-protective responses and suppression mechanisms related to host–parasite cross-talk. The mechanisms involved in innate susceptibility/resistance to CE/AE are mostly unknown [9]. However, some components of host innate immune cells (such as natural killer cells (NKs), macrophages, neutrophils, mast cells, basophils, eosinophils and DCs) have previously been identified in CE patients [9, 13, 15]. Cabrera et al. [19] have shown that the presence of innate immunity compounds in the adventitial layer of the echinococcal cyst can lead to the release of reactive species of oxygen (ROS) and nitrogen (RNS), which results in the infertility of hydatid cysts, whereas EgRAD9 may allow preserving the fertility of hydatid cysts in the presence of ROS and RNS through DNA repairing of protoscoleces (PSCs) [19]. Neutrophils and macrophages are the first responders to detect and eliminate parasites, but their natural activities can be prevented by parasite metabolites. Antigen B secreted by *E. granulosus* can interfere with neutrophil activity *via* the elastase secreted by neutrophilic granulocytes and enable the parasite to escape from the host immune response [20]. In addition, AgB can regulate the host immune system by modifying the activity of macrophages and suppressing the production of effective cytokines [21, 22]. Several studies have also been revealed that exposure to parasite metabolites alters the differentiation, maturity and function of DCs and NKs, which subsequently increases the development of CE and AE [23–26]. DCs anergy against excretory/secretory (E/S) antigens of CE suggests that E/S antigens are immunosuppressive [14]. It is worth noting that eosinophils as effector cells are potentially effective in innate immunity against the metacestode of *E. granulosus*; however, they are not active in adults [27]. In addition, CE patients have been shown to have relatively more NK cells (CD56^+^/CD8^−^) in their peripheral blood mononuclear cells (PBMC) than the control group [28]; however, during the last decade, three innate immune pathways related to the host-*Echinococcus* cross-talk have been characterized, including inflammasomes, TLRs and apoptosis pathways [29–31]. This review presents an update on the latest advances in the knowledge of regulatory functions of innate immune pathways to CE/AE and illustrates how a better understanding of these first responders may lead to an improvement of novel therapeutic vaccines and control strategies. Six bibliographic databases including Science Direct, The Medical Literature Analysis and Retrieval System Online (MEDLINE), PubMed Central (PMC), Scopus, Google Scholar, and ProQuest LLC were searched for articles published between 1997 and 2019, identifying a total of 61 publications. The following MeSH (Medical Subject Headings) keywords were considered in the initial search strategy: “*Echinococcus multilocularis*”, “*Echinococcus granulosus*”, “Innate immunity responses”, “Apoptosis”, “Toll-like receptors”, “Inflammasome”, “siRNA” and “Tumor suppressor-p53”.

## Inflammasome in AE and CE

**Fig. 1:**
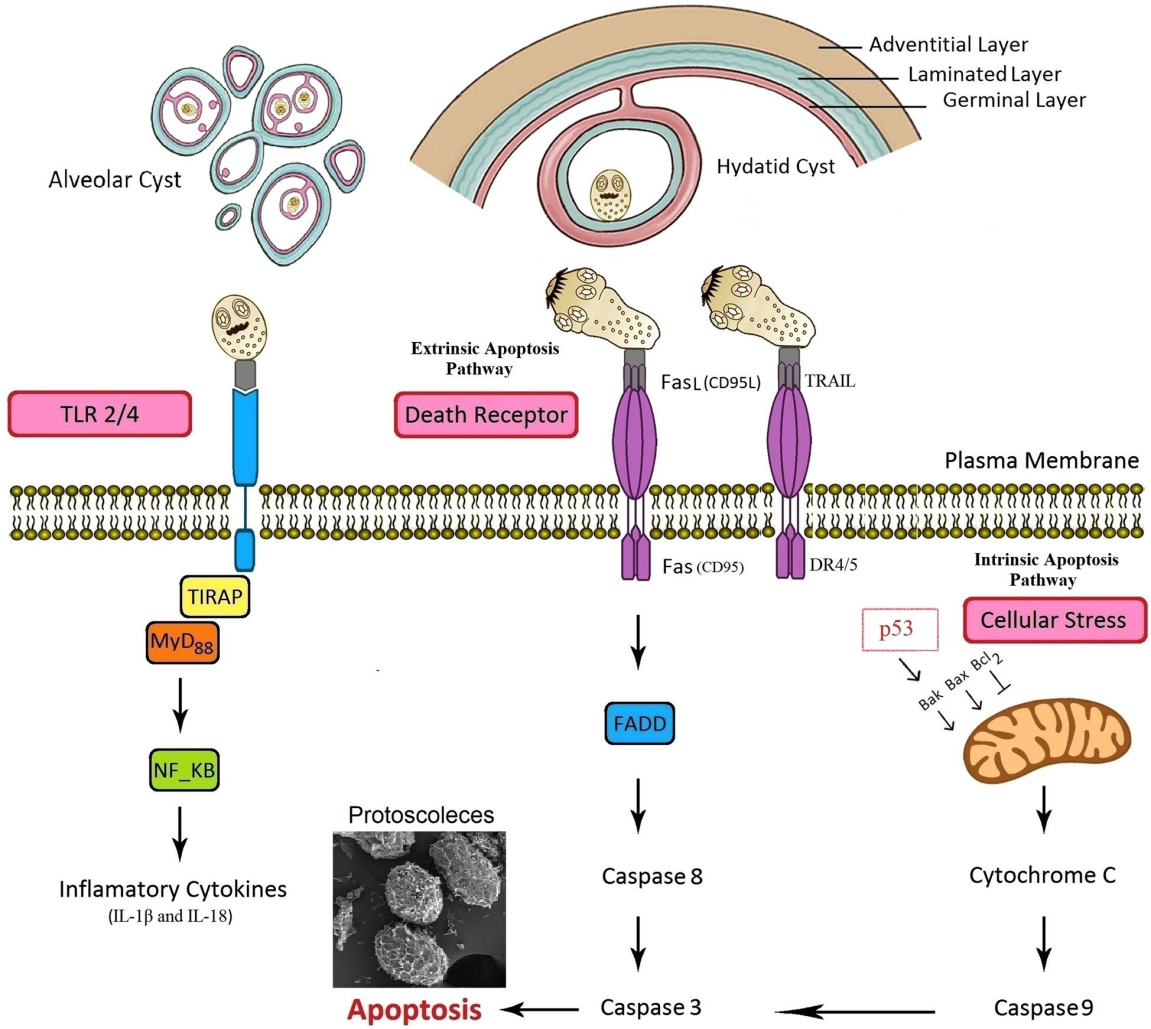
Scheme of identified pathways of host innate immunity (inflammasome, TLRs and apoptosis) against cystic echinococcosis and alveolar echinococcosis in this study

The inflammasome is an intracellular platform containing NLRs family proteins that are highly sensitive to different PAMPs and regulate immune responses through inducing the particular pro-inflammatory cytokines, IL-1β and IL-18, *via* induction of caspase-1 activation [32]. Currently, no adequate data are available regarding the inflammasome activation in CE/AE [13] (Fig. 1). However, similar studies have revealed that some helminth metabolites may activate the inflammasome pathway. *Schistosoma mansoni* and *S. japonicum* soluble schistosomal egg antigens are able to activate the NLRP3 inflammasome and trigger IL-1β secretion in infected mice [30, 33]. In addition, the increased level of IL-18 secretion is related to NLRP3 inflammasome activation in mice and humans infected with the *Trichuris muris*, which results in the suppression of innate and adaptive immune responses [34].

## TLRs in CE and AE

TLRs as one of the PRR subfamilies can recognize various PAMPs derived from pathogens. TLRs are a key stimulator of the host innate immune response through the production of pro-inflammatory cytokines [16, 35]. Currently, thirteen TLRs have identified in mammals, which are typically expressed on the cell surface (TLR1/2/4/5/6/10) or in the endosome membrane (TLR3/7/8/9/11/12/13) of host immune cells [16, 32]. TLRs trigger innate immune signaling by activation of the myeloid differentiation factor 88 (MyD88) and induction of important transcription factors, such as nuclear factor-κB (NF-κB), interferon regulatory interactions (IRFs) and mitogen-activated protein kinases (MAPKs), to produce pro-inflammatory cytokines [32, 36] (Fig. 1).

However, there is limited information on the effects of TLRs in response to *E. granulosus* and *E. multilocularis* infections. Preliminary evidence suggests that AgB of *E. granulosus* can adjust the maturation of DCs through TLRs [24]. Few studies have revealed a significant relationship between high expression levels of TLR2, TLR4 and the serum level of IL-10 during chronic CE infection, indicating the probable functions of TLR2/TLR4 in the process of immune tolerance [37, 38]. An *in vivo* investigation has shown that increased levels of TLR2 and TLR4 mRNA expression and related cytokines (IFN-γ, IL-5, IL-23 and IL-10) in hepatic AE patients protect the parasite from host immunity [39]. In addition, *E. multilocularis* vesicular fluid (Em-VF) affects the differentiation and maturation of monocyte-derived DC. Furthermore, co-stimulation of lipooligosaccharide (LPS) with TLR agonists reinforces the response of healthy blood donors exposed to Em-VF [40]. The high expression of TLR2 and IL-23 is possibly effective in modulating tissue infiltrative growth in CE and AE and its sustainability in the host body [35].

Besides, it has been shown that TLR antagonists or negative regulator agonists can play a key role in treatment, or as an auxiliary vaccine to increase the immune response against parasites [41]. Recent findings have indicated that the E/S products of *E. granulosus* PSCs stimulate B cells to secrete IL-10 *via* TLR-2 signaling [42]. On the one hand, our findings have recently indicated an interesting association between TLR2 and TLR4polymorphisms and their susceptibility to chronic CE [29]. The assessment of TLR4 Asp299Gly and Thr399Ile polymorphisms in patients with recurrent hydatidosis (RH), acute hydatidosis (AH) and healthy groups indicated that the A/G genotype and mutant allele G of TLR4 Asp299Gly have a tendency to be associated with the occurrence of RH and conferred a 3-fold risk for CE susceptibility, whereas the TLR4 Thr399Ile haplotype has been observed only in a patient with pulmonary hydatidosis [29]. In addition, the homozygous mutant-type TLR2Arg753Gln haplotype (Gln/Gln; A/A) has been indicated to be strongly associated with the occurrence of RH (*Pv*: 0.04), conferring a 9-fold increased risk of susceptibility [43]. The mutant allele A of TLR2Arg753Gln has been shown to be a strong risk factor for susceptibility to RH [43].

## Apoptosis in CE and AE

Apoptosis is a programmed cell death that is finely tuned according to the host–parasite interaction [31]. Previously, it has been proven that the apoptotic process is reversed by anti-apoptotic drugs leading to the rescue of the host cells [44]. Understanding of these immunological events is necessary for the development of therapeutic and diagnostic strategies or designing the new generation of vaccines, to prevent the clinical progression of AE/CE. Apoptosis is characterized by morphological and biochemical mechanisms [45]. During apoptosis, the observed morphological changes include DNA fragmentation, chromatin condensation, cell shrinkage and apoptotic bodies [45]. Several intracellular signaling pathways result in the recruitment and activation of a set of proteases known as caspases. The process of apoptosis in CE is known to occur through the two main pathways: the extrinsic pathway (death receptor-mediated pathway) and the intrinsic pathway (mitochondrial pathway). The B-cell lymphoma 2 (Bcl-2) family maintains the mitochondrial pathway through anti-apoptosis proteins (Bcl-2, Bcl-XL, Bcl-w, Bfl-1, Brag-1and Mcl-1) and pro-apoptosis proteins (Bax, Bak, Bad and Bcl-Xs). The extrinsic pathway is triggered by death receptors and their appropriate ligands, including, Fas-L/FasR, TNF-α/TNFR and TRAIL/TRAIL-R [31, 46].

To date, several studies have been conducted on the prominent role of apoptosis in the development or suppression of CE and AE. According to Paredes et al. [47], analyses of DNA fragmentation and caspase-3 activity in the germinal layer confirm that apoptosis has a negative regulatory effect on the generation of PSCs and leads to possible infertility of hydatid cysts [47]. In this respect, high-level expression of apoptosis-inducing ligands, TRAIL and Fas-L, on the surface of the germinal layer of infertile cysts compared to the fertile cysts and healthy host tissue indicates that apoptosis probably plays an important role in infertility of fertile cysts [31]. An *in vivo* study has documented that the expression of anti-apoptotic and proliferation factors in the liver of infected BALB/c mice with *E. multilocularis* can lead to the survival of AE in host’s hepatocytes [48]. Our previous data have indicated that an increased apoptosis of host’s lymphocytes by hydatid cyst fluid (HCF) antigens through the mitochondrial apoptotic pathway is associated with a high expression of Bax and caspase-3 [31, 49]. Furthermore, the reduction of the expression level of Bcl-2 (an anti-apoptotic molecule) in fertile echinococcal cysts makes the parasite able to escape from the host immune system [31, 49] (Fig. 1). Likewise, high expression of TNF-α protein may be associated with apoptosis of monocytes, which can prevent immune response to the *E. multilocularis* infection [50].

An *in vitro* study has shown the strong induction of apoptosis in host’s DCs by E/S-products in early stages (oncosphere) of *E. multilocularis* infection, indicating that the induction of CD4+, CD25+, Foxp3+ and T cells play a crucial role in parasite resistance in chronic echinococcosis [51]. In addition, a study on the relationship between AE infection and apoptosis of CD4+ T lymphocytes in infected mice has indicated that the expression of Bcl-2, C myc, TGF-β and apoptosis of CD+4 T cells can be significantly increased 25 weeks after infection [52], accordingly, the reduction in suppressor signals is probably due to the apoptosis of CD4+ T lymphocytes. In contrast, HCF can increase the proliferation and prohibit the apoptosis of melanoma A375 cells through upregulating the procaspase-3 and Bcl-2 protein as well as downregulating the pro-apoptotic protein Bax [53]. In addition to apoptotic properties of *E. granulosus/E. multilocularis* metabolites on host’s cells, the apoptotic effects of some chemotherapeutic drugs (dexamethasone (DMZ), praziquantel and ABZ sulfoxide (ABZs)) and nanocompounds have also been reciprocally evaluated against microcysts and/or PSCs *in vitro* and *in vivo* settings [54–58].

Hu et al. [54] have shown that H_2_O_2_ and DMZ can stimulate PSCs cell apoptosis, indicating the presence of an apoptotic gene, like CED-3 in PSCs. Naseri et al. [56] have also reported that the caspase-3 mRNA expression was higher in both PSCs treated with ABZs as well as PSCs treated with ABZs-loaded PLGA-PEG compared to the control groups (*P* < 0.05). An investigation on the cloned calmodulin of *E. granulosus* (rEgCaM) has shown that the expression level of rEgCaM increased at the beginning of exposure to H_2_O_2_, but it gradually decreased due to the increased apoptosis of PSCs [59]. The alcoholic extract of *Myrtus communis* can activate apoptosis in hydatid cyst PSCs through the intrinsic pathway by increasing caspases-3 and caspase-9 mRNA expression [60]. A recent study has revealed that the apoptotic effects of arsenic trioxide (As_2_O_3_) on *E. granulosus* PSCs are associated with the elevation of reactive oxygen species levels, disruption of intracellular Ca^2+^ homeostasis, and endoplasmic reticulum stress [52]. The apoptotic properties of gamma rays on *E. granulosus* metacestode showed that caspase-3 activity was higher in the irradiated group compared to the control group [61]. Accordingly, we can infer that apoptosis can reciprocally exert a bi-functional effect on the drug-*Echinococcus* parasite as well as the host-*Echinococcus* metabolite relationship in suppressive and survival mechanisms of the parasite, respectively.

## Can siRNA and anti-apoptotic adjuvants be regarded as therapeutic opportunities for future treatment of CE?

**Fig. 2:**
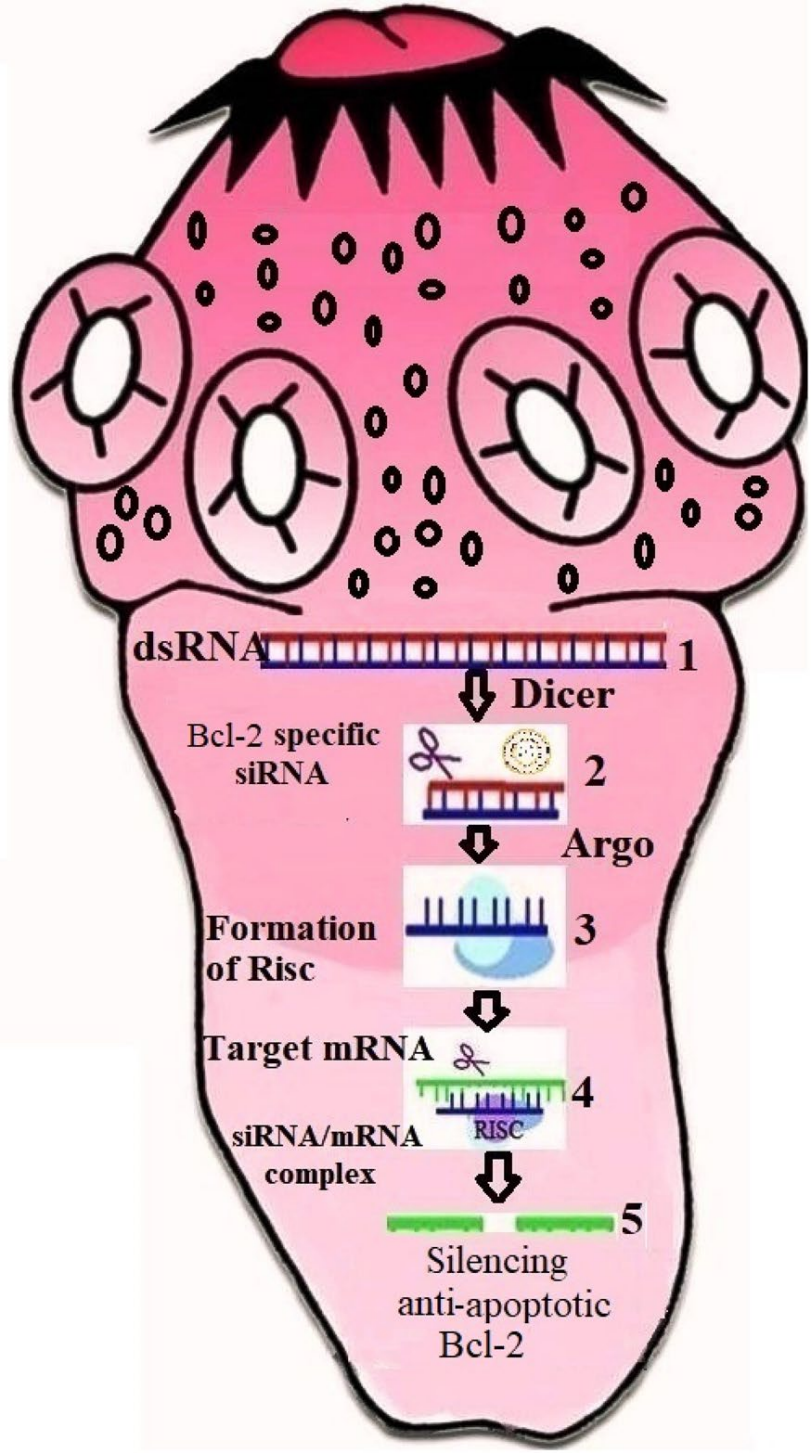
Bcl-2-specific siRNA/mRNA pathway schematization. dsRNA molecules are cleaved by the nuclease Dicer (**1**) and converted to siRNA (**2**). Bcl-2-specific siRNA incorporated into a multiprotein RNA-inducing silencing complex (RISC) (**3**). The RISC attached to complementary target mRNA (**4**) and subsequent endonucleolytic cleavage and gene silencing (**5**)

One of the most imperative approaches in biology is the discovery of small interfering RNA (siRNA), which is able to knockdown and/or downregulate the expression of apoptotic genomes by the mechanism of RNA interference (RNAi). During the RNAi, mRNA degradation is stimulated by complementary siRNA (non-translated double-stranded RNA) using soaking, electroporation and microinjection. RNAi has recently been applied to silence different helminth genomes, including tapeworms, trematodes and nematodes [62]. It has been suggested that the mechanism of specific suppression of an anti-apoptotic gene by the RNAi pathway can be considered as a promising adjunctive treatment in cancer patients [63]. Accordingly, we postulate that the Bcl-2-specific siRNA for silencing of the anti-apoptotic Bcl-2 molecule can upregulate pro-apoptotic Bax expression, which may lead to apoptosis (suppression) of hydatid cyst growth in a susceptible host (Fig. 2). So far, few studies have shown various outcomes regarding using anti-apoptotic interventions during parasitic infections [64]. An *in vivo* experimental study has revealed that transgenic mice experimentally infected with cerebral *Plasmodium falciparum* following treatment with z-VAD (a pan-caspase inhibitor) and also over-expression of Bcl-2, failed to reduce mortality in the infected mice [64]. In contrast, the protective effects of z-VAD indicated that the mice experimentally infected with *Entamoeba histolytica* showed a reduction in mean liver size by 70% compared to the healthy controls [65]. The neuroprotective evaluation of erythropoietin (Epo) in a murine model of cerebral malaria showed that the artesunate-Epo combination can lead to an enhanced survival time than artesunate treatment *via* reducing pro-inflammatory cytokines (IL-8, IL-1ß and TNF-α) [66]. In general, these new therapeutic opportunities have provided reasonable evidence regarding the regulatory role of inhibitors of apoptotic proteins through the development of *E. granulosus* [67, 68].

## Tumor suppressor-p53 in CE and AE

Tumor suppressor-p53 is an essential transcription factor that plays a pivotal role in cell cycle arrest, triggering apoptosis and responding to various genotoxic stresses [69]. Accordingly, investigation of post-immunization apoptotic changes in experimental hydatidosis has shown that the mRNA expression of caspase-3 and p53 were significantly higher in the group immunized with a crude antigen compared to the purified antigen and non-immunized control groups, which caused fewer and smaller cystic lesions [70] (Fig. 1). It has been revealed that the mRNA expression of Emp53 of *E. multilocularis* is a structural and functional homolog of mammalian tumor suppressor p53, which plays a crucial role in response to DNA damage and apoptosis, saving the parasite from oxidative stress effects [71].

## Conclusions

In conclusion, although the generation of chemotherapeutic drugs against CE/AE infections has improved the clinical treatment of these cestodes, the lack of successful control campaigns, including the EG95 vaccine at the continental level indicates the importance of generating novel therapies. Based on the current knowledge, further studies are needed to determine whether the orchestrated *in silico* strategy for designing inhibitors and siRNA against anti-apoptotic proteins and TLRs would be effective to improve the new treatments and therapeutic vaccines against the *E. granulosus* and *E. multilocularis.* Therefore, it is essential to continue exploring how the innate immune mechanisms, associated with the host-*Echinococcus*, interplay promote and/or suppress each other to provide future interventions to treat these neglected infections.

## Data Availability

Data supporting the conclusions of this article are included within the article.

## Abbreviations

CE: cystic echinococcosis
AE: alveolar echinococcosis
siRNA: interfering RNA
NKs: natural killer cells
TLRs: toll-like receptors
Em-VF: *E. multilocularis* vesicular fluid
NF-Κb: nuclear factor-κB
DMZ: dexamethasone
ABZs: ABZ sulfoxide
